# Pulsatile varicose veins: an uncommon presentation of a common condition

**DOI:** 10.1590/1677-5449.210075

**Published:** 2021-08-06

**Authors:** Thilina Gunawardena, Manujaya Godakandage, Balasubramaniyam Saseekaran, Rezni Cassim, Mandika Wijeyaratne

**Affiliations:** 1 The Royal Liverpool University Hospital, Liverpool, United Kingdom.; 2 National Hospital of Sri Lanka, Colombo, Sri Lanka.; 3 Royal Free Hospital, London, United Kingdom.

**Keywords:** pulsatile varicose veins, Parkes Weber syndrome, tri-cuspid regurgitation, secondary varicose veins, varizes pulsáteis, síndrome de Parkes Weber, regurgitação tricúspide, varizes secundárias

## Abstract

Varicose veins of the lower limbs are common. However, pulsatile varicose veins are unusual. They could be an indicator of a sinister underlying pathology, such as severe cardiac dysfunction. It is easy to miss these rare cases during clinical workup, which can result in futile treatment with potentially dangerous consequences. In this report, we describe 2 cases of pulsatile varicose veins that highlight different etiologies and management strategies for this condition.

## Resumo

Varizes dos membros inferiores são comuns. Entretanto, varizes pulsáteis são raras, podendo ser indicadoras de uma patologia subjacente sombria como disfunção cardíaca grave. É fácil deixar passar esses casos raros durante exames clínicos, o que pode resultar em tratamento fútil com consequências potencialmente perigosas. Neste relato, descrevemos dois casos de varizes pulsáteis que evidenciam as diferentes etiologias e estratégias de manejo para essa condição.

## INTRODUCTION

Dilated, tortuous, superficial veins of the lower limbs are extremely common. The majority of these varicose veins (VV) are classified as primary VV. They are a result of junctional incompetence, structural defects in superficial vein trunks, or reflux in the perforator veins. In contrast, secondary VV are relatively uncommon. They can be caused by pathologies such as deep vein obstruction or reflux due to previous deep vein thrombosis, compression from pelvic tumors, and abnormal arteriovenous communications.^1^ Rarely, secondary VV can be pulsatile. It is important not to miss these infrequent cases of pulsatile VV, because the approach to their diagnosis and treatment is markedly different. Here we describe 2 patients who presented with unilateral lower limb VV, which were found to be pulsatile on close examination. We believe that these 2 cases highlight the spectrum of underlying etiologies for this peculiar presentation of a common condition.

The study was conducted in accordance to the relevant standards of the institutional ethics committee and the Helsinki declaration. Informed, written consent was obtained from both patients prior to data collection and publication of the case reports.

## CASE REPORTS

### Case 1

A 77 year old female patient was referred to the vascular surgery clinic for further evaluation of a possible vascular malformation at the right lower thigh region. She was a diabetic patient and had a history of rheumatic mitral valve stenosis. She had undergone mitral valve replacement in 2005 and since then was on warfarin. In 2010 she noted abnormal, dilated veins over the right lower thigh region, but as they were asymptomatic, she chose not to seek medical attention. When the right leg developed progressive swelling with some discomfort she brought the matter to the attention of doctors in 2019. At the first contact surgical clinic, a duplex scan was done to exclude deep vein thrombosis. Suspecting a vascular anomaly they next arranged a contrast CT of the lower limb, which failed to demonstrate an arteriovenous malformation (AVM) or a vascular tumor.

**Figure 1:**
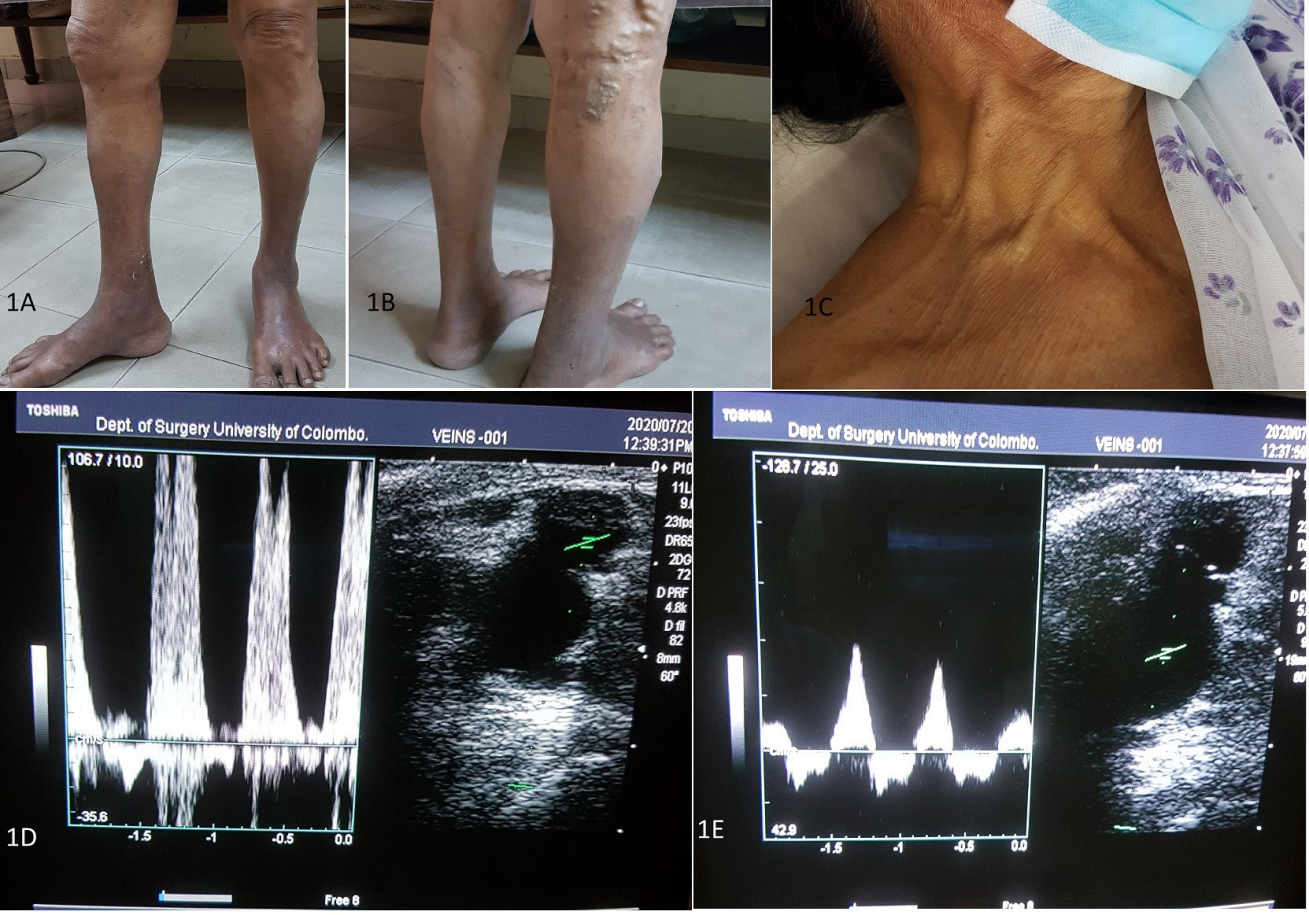
(A), (B) Right lower limb VV; (C) engorged neck veins; (D) pulsatile flow in the GSV at the SFJ; (E) pulsatile flow in the femoral vein.

When we saw her at our clinic, the first striking feature we noted was the abnormal distribution pattern of VV (Figures 1A, B). There was a bunch of dilated tortuous veins at the medial aspect of the mid-thigh as well as the posterior aspect of the lower thigh of the right lower limb. The veins over the medial thigh communicated with the dilated proximal greater saphenous vein (GSV), which was pulsatile. A bruit was audible over the saphenofemoral junction (SFJ). When the limb was elevated the veins failed to empty completely. Pedal pulses were present and there was pitting ankle edema. On the contralateral lower limb, a few varicosities were noted and there was no associated edema. Auscultation of the precordium revealed a pansystolic murmur best heard at the left lower sternal edge. The neck veins were dilated (Figure 1C). The abdomen was mildly distended. We could not demonstrate a pulsatile, enlarged liver.

A venous duplex scan of her right lower limb revealed arterial type pulsatile flow in the proximal GSV (Figure 1D) and the common femoral vein (Figure 1E). The abnormal VV also had a pulsatile flow pattern. There was saphenofemoral incompetency with reflux in the GSV and the abnormal superficial veins.

Given the clinical features and the history of rheumatic fever, we suspected she may have tricuspid regurgitation (TR). The 2D echocardiogram confirmed severe TR with moderate pulmonary hypertension. The pressure gradient across the tricuspid valve (TV) was 40mmHg. Considering the age of the patient and the prior history of open heart surgery, a joint decision was taken by the cardiologists and the vascular surgeons to manage the tricuspid incompetence and the VV conservatively. Presently she is doing well, with the use of Class II below-knee compression stockings.

### Case 2

**Figure 2:**
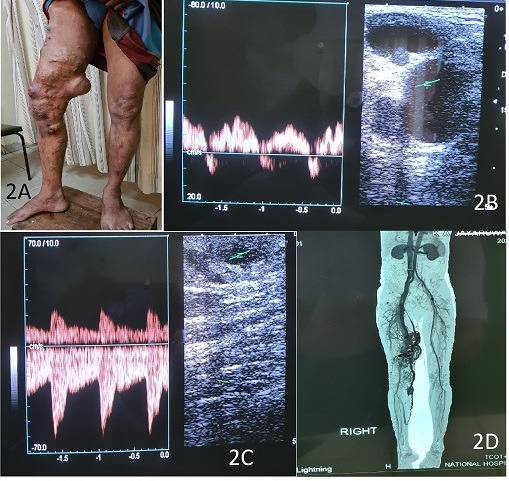
(A) Abnormal right lower limb VV; (B) arterial type flow at the SFJ; and (C) superficial veins; (D) CT angiogram depicting the AVM.

A 47-year-old male patient presented to the vascular surgery clinic with recurrent bleeding from large varicose veins over the right thigh. He had a history of sclerotherapy for VV of the same limb and had undergone ipsilateral saphenofemoral ligation (SFL) and stripping at a very young age. On examination, there were large varicosities over the right thigh and leg with an abnormal distribution pattern (Figure 2A). The circumference of the right thigh was significantly larger compared to the contralateral limb. However, there was no limb length discrepancy. Ulceration was noted on the right thigh, corresponding to a recent bleeding point. There were no VV over the opposite limb. On palpation, the dilated, massive veins were pulsatile. They failed to empty with limb elevation. Pulse examination detected no abnormalities. Pre-cordial examination for murmurs was negative. A duplex scan of the right lower limb showed an arterial type of flow pattern in the superficial varices (Figure 2B, C) and the femoral vein. A CT angiogram of the lower limbs demonstrated a large AVM involving the right thigh and the leg. (Figure 2D) The main feeding vessels were from the superficial femoral artery and the popliteal artery. A clinical picture was suggestive of Parkes-Weber syndrome (PWS). Considering recurrent bleeds from the VV, the patient was referred for angioembolization of the AVM.

## DISCUSSION

Pulsatile varicose veins are extremely rare. Severe TR and AVMs are the predisposing causes described in the literature.^2^^,^^3^ Unless extreme care is taken, it is easy to miss a case of pulsatile varicose veins and a treatment approach similar to a case of uncomplicated, primary varicose veins can result in disastrous consequences.^1^ A case has been reported in which SFL was attempted in a patient with varicose veins caused by severe TR and the surgeon had to abandon it because of the friable nature of the veins and the resultant haemorrhage.^4^ Another interesting vignette by Klein et al. reports a patient with pulsatile VV due to undiagnosed TR, who was subjected to unnecessary aortic exploration looking for an arterio-venous communication.^5^

In severe TR, the regurgitating blood through the incompetent TV sets up a venous pressure wave which is transmitted along the inferior vena cava and down the lower limb deep and superficial veins.^2^^,^^3^ Although transmission of venous pressure occurs bilaterally, some patients can have the VV confined to a single limb.^6^ The venous valve dysfunction in these patients may be secondary to the severe TR, or VV caused by primary valve dysfunction can progress due to the high venous pressures. Some of these patients may have a history of femoral vein cannulation for cardiac catheterization or cardiopulmonary bypass, so it is important to exclude a potential arteriovenous fistula.^3^ A simple test to differentiate TR from arteriovenous fistulae in this situation is to occlude to SFJ with a finger and observe for the disappearance of pulsatility in the VV. If this sign is positive, then the possibility of arterio-venous communication is unlikely.^6^

Another physical finding that may point towards the presence of an arteriovenous fistula is the Nicoladoni-Branham sign or slowing of the heart rate upon compression of the inflow to the fistula.^7^

There is an inclination for conservative management of pulsatile VV secondary to TR, as the patients affected tend to be elderly with significant cardiac comorbidities.^2^ However, there are cases where SFL and GSV laser ablation have been utilized as treatment options with satisfactory outcomes.^2^^,^^6^

PWS is a congenital disorder characterized by high-flow AVMs and limb hypertrophy. Bleeding from VV is a rare presentation of this syndrome. The disorder generally tends to affect a single extremity. The limb asymmetry can be a discrepancy in the length due to bony overgrowth or an increase in limb circumference as a result of soft tissue hypertrophy.^7^ This second phenomenon was seen in our patient.

VV in PWS is a consequence of hyperdynamic flow caused by the AVMs.^8^ Failure to identify the correct etiology in our patient who presented with VV at a very young age led to futile therapy with sclerotherapy and SFL and stripping of the GSV. As a result, for several years he suffered recurrent bleeds from engorged superficial veins.

Treatment for PWS is indicated when there are symptoms, functional impairment or cosmetic concerns. At present, transarterial embolization of AVMs is an increasingly used treatment option. Sometimes more than one treatment session may be required to achieve desired outcomes.^9^ Surgery also has a secondary role and excision of AVMs, corrective surgery for limb hypertrophy, and amputation for intractable symptoms have been used as options.^8^

## CONCLUSIONS

Pulsatile VV are rare and it is easy to miss the diagnosis. An abnormal distribution pattern in the VV should alert the physician to the possibility of secondary causes. The etiology of the pulsatility of the veins should be confirmed by clinical and radiological methods before embarking on treatment, which should be individualized for each case.

## References

[1] Li X, Feng Y, Liu Y, Zhang F (2019). Varicose veins of the lower extremity secondary to tricuspid regurgitation. Ann Vasc Surg.

[2] Chihara S, Sawada K, Tomoeda H, Aoyagi S (2017). Pulsatile varicose veins secondary to severe tricuspid regurgitation: report of a case successfully managed by endovenous laser treatment. Ann Vasc Surg.

[3] Dayantas J, Liatas AC, Lazarides M (1990). Pulsatile varicose veins caused by tricuspid valve regurgitation. Phlebology.

[4] Badger SA, Makar RR, Chew EW, Lee B (2012). Recurrent bilateral varicose veins secondary to tricuspid regurgitation. Ir J Med Sci.

[5] Klein HO, Shachor D, Schneider N, David D (1993). Unilateral pulsatile varicose veins from tricuspid regurgitation. Am J Cardiol.

[6] Abbas M, Hamilton M, Yahya M, Mwipatayi P, Sieunarine K (2006). Pulsating varicose veins!! The diagnosis lies in the heart. ANZ J Surg.

[7] Burchell HB (1958). Observations on bradycardia produced by occlusion of an artery proximal to an arteriovenous fistula (Nicoladoni-Branham sign). Med Clin North Am.

[8] Girón-Vallejo O, López-Gutiérrez JC, Fernández-Pineda I (2013). Diagnosis and treatment of Parkes Weber syndrome: a review of 10 consecutive patients. Ann Vasc Surg.

[9] Banzic I, Brankovic M, Maksimović Ž, Davidović L, Marković M, Rančić Z (2017). Parkes Weber syndrome-Diagnostic and management paradigms: A systematic review. Phlebology.

